# Distinct roles for major and minor antigen barriers in chimerism‐based tolerance under irradiation‐free conditions

**DOI:** 10.1111/ajt.16177

**Published:** 2020-07-24

**Authors:** Benedikt Mahr, Nina Pilat, Nicolas Granofszky, Moritz Muckenhuber, Lukas W. Unger, Anna M. Weijler, Mario Wiletel, Romy Steiner, Lisa Dorner, Heinz Regele, Thomas Wekerle

**Affiliations:** ^1^ Section of Transplantation Immunology Department of Surgery Medical University of Vienna Vienna Austria; ^2^ Clinical Institute of Pathology Medical University of Vienna Vienna Austria

**Keywords:** basic (laboratory) research/science, immunobiology, tolerance: chimerism, tolerance: costimulation blockade, tolerance: experimental, tolerance: mechanisms

## Abstract

Eliminating cytoreductive conditioning from chimerism‐based tolerance protocols would facilitate clinical translation. Here we investigated the impact of major histocompatibility complex (MHC) and minor histocompatibility antigen (MiHA) barriers on mechanisms of tolerance and rejection in this setting. Transient depletion of natural killer (NK) cells at the time of bone marrow (BM) transplantation (BMT) (20 × 10^6^ BALB/c BM cells → C57BL/6 recipients under costimulation blockade [CB] and rapamycin) prevented BM rejection. Despite persistent levels of mixed chimerism, BMT recipients gradually rejected skin grafts from the same donor strain. Extending NK cell depletion did not improve skin graft survival. However, F1 (C57BL/6×BALB/c) donors, which do not elicit NK cell‐mediated rejection, induced durable chimerism and tolerance. In contrast, if F1 donors with BALB/c background only were used (BALB/c×BALB.B), no tolerance was observed. In the absence of MiHA disparities (B10.D2 donors, MHC‐mismatch only), temporal NK cell depletion established stable chimerism and tolerance. Conversely, MHC identical BM (BALB.B donors, MiHA mismatch only) readily engrafted without NK cell depletion but no skin graft tolerance ensued. Therefore, we conclude that under CB and rapamycin, MHC disparities provoke NK cell‐mediated BM rejection in nonirradiated recipients whereas MiHA disparities do not prevent BM engraftment but impede skin graft tolerance in established mixed chimeras.

## Graphical abstract

This article shows that in nonirradiated mice, major histocompatibility antigen barriers are mainly responsible for NK cell–mediated bone marrow rejection under costimulation blockade and rapamycin while minor histocompatibility antigen barriers determine donor skin graft survival. See Sánchez‐Fueyo and Dazzi's editorial on page 919.

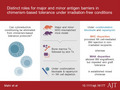

**Abbreviations**
BM: bone marrow
BMT: bone marrow transplantation
CB: costimulation blockade
MiHA: minor histocompatibility antigens
MLR: mixed lymphocyte reaction
MST: median survival time
NK: natural killer
Rapa: rapamycin
SD: standard deviation

## 1 INTRODUCTION

Transplantation brings great relief for patients with end‐stage organ failure by improving their survival and quality of life.^1^ These improvements, however, are taking a toll in the form of constant immunosuppression, which predisposes the patients to severe infections and malignant diseases. Besides, common immunosuppressive medications entail additional undesired effects, such as nephrotoxicity, and may not constitute a durable solution because long‐term allograft survival is still limited by chronic rejection.^2^, ^3^ These and other caveats still fuel the dream for a state in which the patients indefinitely retain an allograft without requiring any immunosuppressive medication, namely transplantation tolerance. Several approaches have been developed in the murine setting to achieve this desired state from which the induction of mixed chimerism emerged as a promising strategy.^4^ Furthermore, it has been the only approach that has already been successfully translated in several independent clinical trials.^5^, ^6^, ^7^

In the mid‐1950s, it was discovered that transplanting allogeneic bone marrow (BM) into lethally irradiated mice can confer tolerance to solid tissues from the same donor.^8^ Even back then, the irradiation required for successful BM engraftment in adult recipients raised serious concerns about clinical implementation.^9^ It took 30 years until it was realized that replacing only a part of the recipient's BM is sufficient to achieve tolerance^10^— a state referred to as mixed chimerism. The low irradiation doses necessary to induce mixed chimerism provided a reasonable basis for clinical implementation.^6^ Further progress could be achieved through the increased understanding of T cell activation and allorecognition, which led to the provision of new therapeutic possibilities. T cell–depleting antibodies broke the first ground^11^ but it was the specific blockade of T cell costimulatory pathways that allowed the avoidance of global depletion of the recipient T cell repertoire. In particular, the concomitant use of CTLA4‐Ig and α‐CD40L proved to be highly effective^12^ especially when combined with the mammalian target of rapamycin inhibitor rapamycin.^13^ But even then, allogeneic BM was rejected unless low doses of total body irradiation (TBI) were applied^14^ or unrealistically high BM doses were used.^15^, ^16^ For clinical implementation, it would be advantageous, if conventional doses of BM could be transplanted without any cytotoxic treatment. Eliminating the cytotoxic preconditioning is, however, a two‐edged sword. The lower the cytotoxic treatment, the greater the resistance of the immune system against the allogeneic cells. Because the individual leukocyte populations have different sensitivities to radiation,^17^ it remains to be delineated which mechanisms of rejection prevail in the nonirradiated immune system.

In this regard, we could recently show that transient natural killer (NK) cell depletion at the time of bone marrow transplantation (BMT) can obviate the need for any irradiation when conventional doses of fully mismatched BM (BALB/c → C57BL/6) are transplanted under the cover of CB and rapamycin.^18^ The recipient mice developed durable levels of mixed chimerism but skin allografts from the same donor were gradually rejected. To elucidate the contribution of NK cells to these unexpected results, we used F1 mice as BM donors (CB6F1) as they are not targeted by recipient NK cells. CB6F1 mice constitute the first generation of offspring from C57BL/6 (H2^b^) recipients and BALB/c (H2^d^) donors and thus concomitantly express paternal and maternal major histocompatibility complex (MHC) molecules (H2^b/d^). BALB/c MHC molecules expressed on CB6F1 tissues provoke T cell alloresponses in C57BL/6 recipients while at the same time C57BL/6 MHC molecules prevent donor cells from missing self‐recognition by recipient NK cells. Replacing BALB/c donors by CB6F1 mice resulted in durable chimerism and robust skin graft tolerance.^18^ These data strongly suggest NK cells as main barrier for BM engraftment under CB and rapamycin but also raise new questions about the mechanisms preserving skin allografts. The aim of this study was to identify those factors impeding skin graft tolerance in NK cell‐depleted recipients conditioned solely with CB and rapamycin.

## 2 MATERIAL AND METHODS

### 2.1 Mice

C57BL/6, BALB/c, and CB6F1 (F1) mice were purchased from Charles River (Sulzfeld, Germany). B6.SJL‐Ptprca Pepcb/BoyJ (CD45.1), C.B10‐H2b/LilMcdJ (BALB.B), and B10.D2‐Hc1H2dH2‐T18c/nSnJ (B10.D2) mice were obtained from Jackson Laboratory and bred at the Department of Biomedical Research, Medical University of Vienna (Austria). F1.BALB/c mice were obtained by crossing BALB/c females with BALB.B males. All mice were housed under specific pathogen‐free conditions, and female mice were used between 8 and 10 weeks of age with a body weight between 18 and 22 g. Up to 5 animals were kept in individually ventilated polysulfone cages (Tecniplast, Buguggiate, Italy) at a monitored temperature of 20‐24°C, with humidity between 50% and 70%, a constant 12 hours light/dark cycle, and at least 70 air changes per hour. The cages were bedded with decorticated aspen wood and enriched with nesting material (Abedd). Animals were provided with sterilized water and rodent chow (Sniff) ad libitum. All surgeries were performed under general anesthesia using a mixture of ketamine (100 mg/kg) and xylazine (5 mg/kg) intraperitoneally. All animals were treated according to European Union guidelines of animal care. All animal experiments were approved by the institutional review board of the Medical University of Vienna and by the Austrian Ministry of Science and Research (permission number GZ: GZ 66.009/0230–II/3b/2011, BMWFW‐66.009/0028‐WF/V/3b/2015).

### 2.2 BMT and antibody treatment

C57BL/6 recipient mice received 20 × 10^6^ unseparated BALB/c, B10.D2, CB6F1, or F1.BALB/c BM cells (d0). CD45.1 recipient mice were transplanted with 20 × 10^6^ unseparated BALB.B BM cells (d0). BM cells were collected from long and hip bones and preserved in M199 medium (Sigma‐Aldrich, St. Louis, MO) supplemented with 4 μg/mL Gentamicin Sulfate (MP Biomedicals, Irvine, CA) and 10 mM Hepes Buffer (MP Biomedicals). All BMT recipients additionally received CB consisting of α‐CD40L (1 mg: d0; clone MR1; Bio X Cell, West Lebanon, NH) and CTLA4‐Ig (0.5 mg: d2; Bristol‐Myers Squibb, New York, NY) and a short course of rapamycin (0.1 mg: d–1, d0, d2; LC Laboratories, Woburn, MA). Selected recipients of BALB/c, BALB.B, or B10.D2 BM additionally received α‐NK1.1 (0.25 mg: clone PK136; Bio X Cell) either at the time of transplantation (d–1, d2, d5, d8, short‐α‐NK1.1) or regularly until the end of follow‐up (d–1, d2, d5, d8, d28, d56, d84, 112, 140, 168) (long‐α‐NK1.1). Antibodies, fusion proteins, and rapamycin were administered intraperitoneally (i.p.). Mixed chimerism was defined as having at least 2 lineages displaying >0.5% donor cells.

### 2.3 Skin transplantation

Full‐thickness tail skin was grafted 4‐6 weeks after BMT, fixed with sutures and protected by bandages for 7 days. Grafts were visually inspected thereafter at short intervals and considered to be rejected when less than 10% remained viable. Mice were anesthetized with ketamine (Ketalar, 100 mg/kg) and xylazin (Rompun, 5 mg/kg). Postoperative analgesia consisted of buprenorphine (Buprenovet, day 0; 0.01‐0.05 mg/kg/d i.p.), followed by piritramide (Dipidolor, 15 mg in 250 mL 0.4% glucose water) in drinking water ad libitum for 1 week.

### 2.4 Mixed lymphocyte reaction

4 × 10^5^ congenic responder splenocytes (C57BL/6, CD45.1) were cultured for 5 days in RPMI 1640 supplemented with 10% FCS (Linaris, Dossenheim, Germany), PenStrep (100 U penicillin, 100 lg streptomycin per milliliter; Sigma‐Aldrich), 10 mM HEPES (MP Biomedicals), 1 mM sodium pyruvate (Sigma‐Aldrich), 1× nonessential amino acids (Sigma‐Aldrich), and 10 µM β‐Mercaptoethanol (Sigma‐Aldrich). CD45.1 responder cells were stimulated with equal numbers of CD45.2^+^ C57BL/6, BALB/c, F1, or F1.BALB/c splenocytes. Proliferation was assessed by flow cytometry by measuring the expression of Ki‐67 in responding (CD45.1) CD4 and CD8 T cells after 5 days of culture.

### 2.5 Flow cytometry

The presence of donor cells was assessed at regular intervals by staining the donor strain‐specific marker (H2‐Dd) on blood leukocytes. Overall chimerism levels were calculated as percentage of H2‐Dd^+^ cells among all CD45^+^ leukocytes. BALB.B donor cells were identified as being CD45.2 positive and overall chimerism was calculated as percentage of CD45.2^+^ cells among all CD45 leukocytes (CD45.1^+^ + CD45.2^+^). Lineage‐specific chimerism was evaluated as percentage of donor cells amidst distinct lineage markers (CD4, CD8, CD19, Mac‐1). Deletion of donor‐reactive CD4 T cells was assessed by measuring the amount of distinct Vβ subsets (ie, Vβ11+ and Vβ5+ T cells recognizing superantigen presented by donor MHC class II [I‐E], not being expressed in B6 mice).^19^ Mac‐1 FITC (M1/70), CD19 PE (6D5), CD8‐PE‐Cy7 (53‐6.7), CD4‐APC (RM4‐5), H2‐Dd‐Bio (34‐2‐12), Vβ5‐APC (MR9‐4), Vβ8.1/V8.2‐PE (MR5‐2), Vβ11‐FITC (KT11), CD122‐PE (5H4), CD49b‐FITC (DX5), Ki‐67‐PE‐Cy7 (SolA15), and SAV‐APC were purchased from BioLegend (San Diego, CA). For intracellular staining the cells were permeabilized with the Foxp3/Transcription Factor Staining Buffer Set from eBioscience (San Diego, CA) according to the manufacturer's specification. Flow cytometric analysis was performed with a BD FACSCanto II flow cytometer and data were analyzed by FlowJo (10.0.8) software (Tree Star, Ashland, OR).

### 2.6 Histology

Grafts were retrieved at the end of the observation period. Samples were fixed in 7.5% formalin overnight. Paraffin blocks were subsequently sectioned and stained with hematoxylin and eosin. Slides were scanned with an Aperio ScanScope scanner (Aperio Technologies, Inc, Vista, CA). The Aperio ScanScope allowed scanning of the whole slide using a 920‐objective lens with a numerical aperture of 0.75 coupled with a double objective to achieve a scan of whole slides at 920 magnification. Digitalized slides were viewed and annotated with an Aperio ImageScope.

### 2.7 Statistics

Data were statistically analyzed with GraphPad Prism 5.0 (Graph Pad Inc, La Jolla, CA). A 2‐sided Student's *t* test with equal variances was used to compare percentages of chimerism levels, Vβ subtypes, and Ki67 expression. Total chimerism levels were compared between groups by using analysis of variance (ANOVA). A *P* value below .05 was considered to denote statistical significance (*P < .05, **P < .01, ***P < .001, ****P < .0001, n.s. *P *> .05).

## 3 RESULTS

### 3.1 Permanent NK cell depletion does not promote skin graft tolerance

**FIGURE 1:**
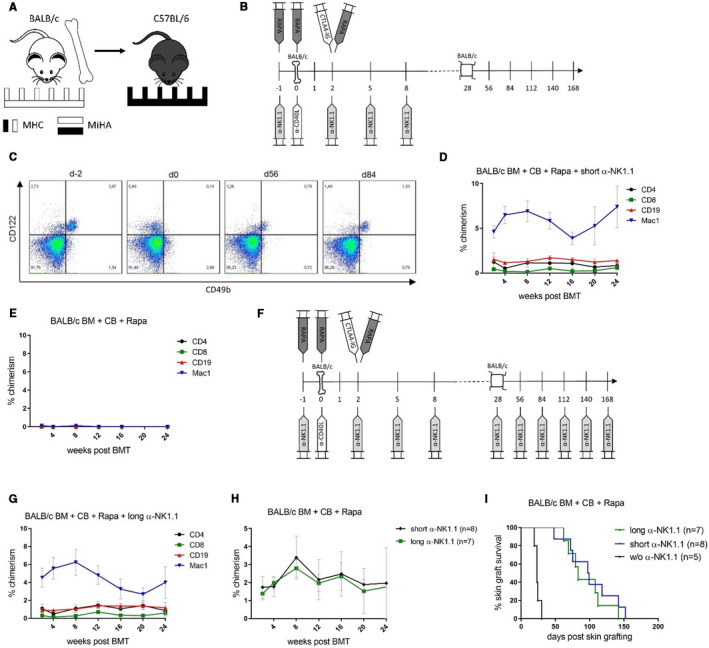
Long‐term natural killer (NK) cell depletion does not promote skin graft survival. C57BL/6 mice received 20 × 10^6^ BALB/c BM under costimulation blockade (CB) and rapamycin. NK cells of selected mice were depleted (α‐NK1.1) at the time of bone marrow transplantation (BMT) (short α‐NK1.1) or permanently (long α‐NK1.1). A, Schematic illustration of the experimental setup outlining the major (MHC) and minor antigen (MiHA) disparities between donor and recipient. B, Schematic illustration outlining the course of the experimental protocol in which NK cells were transiently depleted. C, CD49b^+^ CD122^+^ NK cells disappeared 1 day (0) after the first dose (–1) of transient NK cell depletion from the blood and started to return between 8 and 12 wk later. Representative mouse is shown. D,E, Mean percentages ± SD of donor blood chimerism among indicated lineages is shown over time for recipients of transient NK cell depletion (n = 8) and recipients without NK cell depletion (n = 5). F, Schematic illustration displaying the course of the experimental protocol with permanent NK cell depletion. G, Mean percentages ± SD of donor chimerism of indicated lineages in the blood is shown over time (n = 7). H, Mean percentages ± SD of total donor chimerism in blood is shown over time for indicated treatments. I, Donor (BALB/c) skin graft survival of specified BMT recipients is shown over time [Color figure can be viewed at wileyonlinelibrary.com]

First, we investigated whether NK cells returning after antibody‐mediated depletion could impede BALB/c skin allograft survival. C57BL/6 mice received 20 × 10^6^ unseparated, fully (ie, major [MHC] and minor [MiHA] antigen) mismatched BALB/c BM cells (Figure 1A) together with CB, consisting of α‐CD40L (d0) and CTLA4‐Ig (d2), as well as a short course of rapamycin (d–1, d0, d2), without irradiation. NK cells of recipients were depleted by a monoclonal antibody (α‐NK1.1) at the time of transplantation (d–1, d2, d5, d8) (Figure 1B). NK cells were absent from the blood 1 day after the first dose of α‐NK1.1 and started to return in the blood 8‐12 weeks post‐BMT (Figure 1C).^18^ In accordance with our previous results transient depletion of NK cells around the time of BM infusion induced persistent levels of mixed chimerism in all recipients (8/8) (Figure 1D), whereas chimerism was lost in the absence of NK cell depletion (5/5) (Figure 1E) indicating that NK cells impede BM engraftment under CB and rapamycin. Despite successful BM engraftment, all BMT recipients receiving α‐NK1.1 eventually rejected skin grafts from the same donor strain (median survival time [MST] = 98, *P* = .0001 vs without α‐NK1.1) (Figure 1I). To test the hypothesis that returning NK cells impede skin graft tolerance, we depleted NK cells long‐term by α‐NK1.1 (d‐1, d2, d5, d8, d28, d56,…) (Figure 1F). In the lasting absence of NK cells, chimerism was again achieved (Figure 1G) albeit without any increase in chimerism levels (Figure 1H). Unexpectedly, BALB/c skin graft survival was not extended in comparison to transient NK cell depletion (Figure 1I). Therefore, we conclude that it is not the NK cells returning after temporal depletion that are responsible for provoking (directly or indirectly) skin graft rejection in nonirradiated mice treated with CB and rapamycin.

### 3.2 Minor antigen disparities impede skin graft tolerance in the absence of NK cell alloreactivity

**FIGURE 2:**
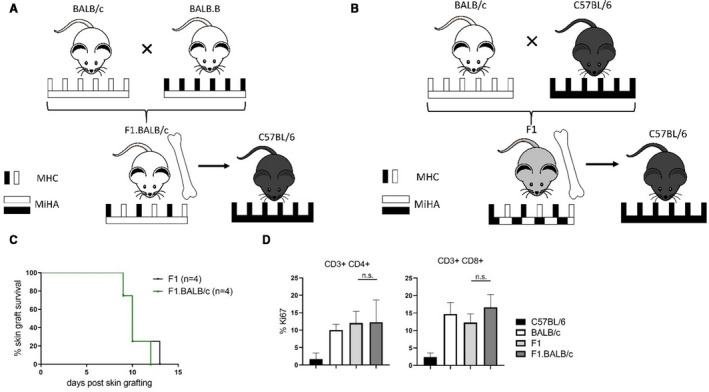
Alloreactivity of F1 mice displaying different minor antigen disparities. The alloreactivity between F1 and F1.BALB/c donor mice was compared in vivo and in vitro. A,B, Schematic illustration of the experimental setup describing the major (MHC) and minor antigen (MiHA) disparities between donor and recipient. C, Naïve C57BL/6 mice received skin allografts from either F1 or F1.BALB/c donors. Skin graft survival of indicated donors is shown over time. D, In vitro proliferation (% Ki‐67) of responding CD45.1^+^ C57BL/6 CD3^+^ CD4^+^ and CD3^+^ CD8^+^ splenocytes stimulated with CD45.2^+^ C57BL/6, BALB/c, F1, or F1.BALB/c splenocytes for 5 d. Bars represent mean ± SD of 3 independent experiments [Color figure can be viewed at wileyonlinelibrary.com]

From previous results we knew that transplantation of F1 BM (triggering no NK alloreactivity) into parental recipients leads to chimerism and permanent acceptance of F1 skin grafts under CB and rapamycin.^18^ Therefore, we hypothesized that tissue‐specific MiHA disparities, which are present in BALB/c but not to the same extent in F1 mice, could account for the gradual rejection of BALB/c skin grafts in mixed chimeras. To test this assumption, we crossed BALB/c females (H2^d^) with BALB.B males. BALB.B mice share all MiHAs with BALB/c donors but express the same MHC molecules as C57BL/6 recipients (H2^b^). The resulting F1 generation (F1.BALB/c) consequently expresses the same MiHAs as BALB/c donors but is resistant to NK cell attack because of the concomitant expression of BALB/c and C57BL/6 MHC molecules (H2^b/d^) (Figure 2A). In comparison, F1 mice express the same constellation of MHC molecules (H2^b/d^) but feature a mixed background of recipient C57BL/6 and donor BALB/c mice (Figure 2B). Because F1.BALB/c mice have not yet been used for tolerance studies, we characterized their alloreactivity in comparison to F1 mice. Naïve C57BL/6 mice rejected F1 and F1.BALB/c skin allografts at the same time (Figure 2C). In an in vitro proliferation assay, comparable numbers of recipient CD4 and CD8 T cells proliferated in response to F1, F1.BALB/c, and BALB/c splenocytes (Figure 2D). These observations indicate that F1 and F1.BALB/c trigger a similar degree of T cell alloreactivity.

**FIGURE 3:**
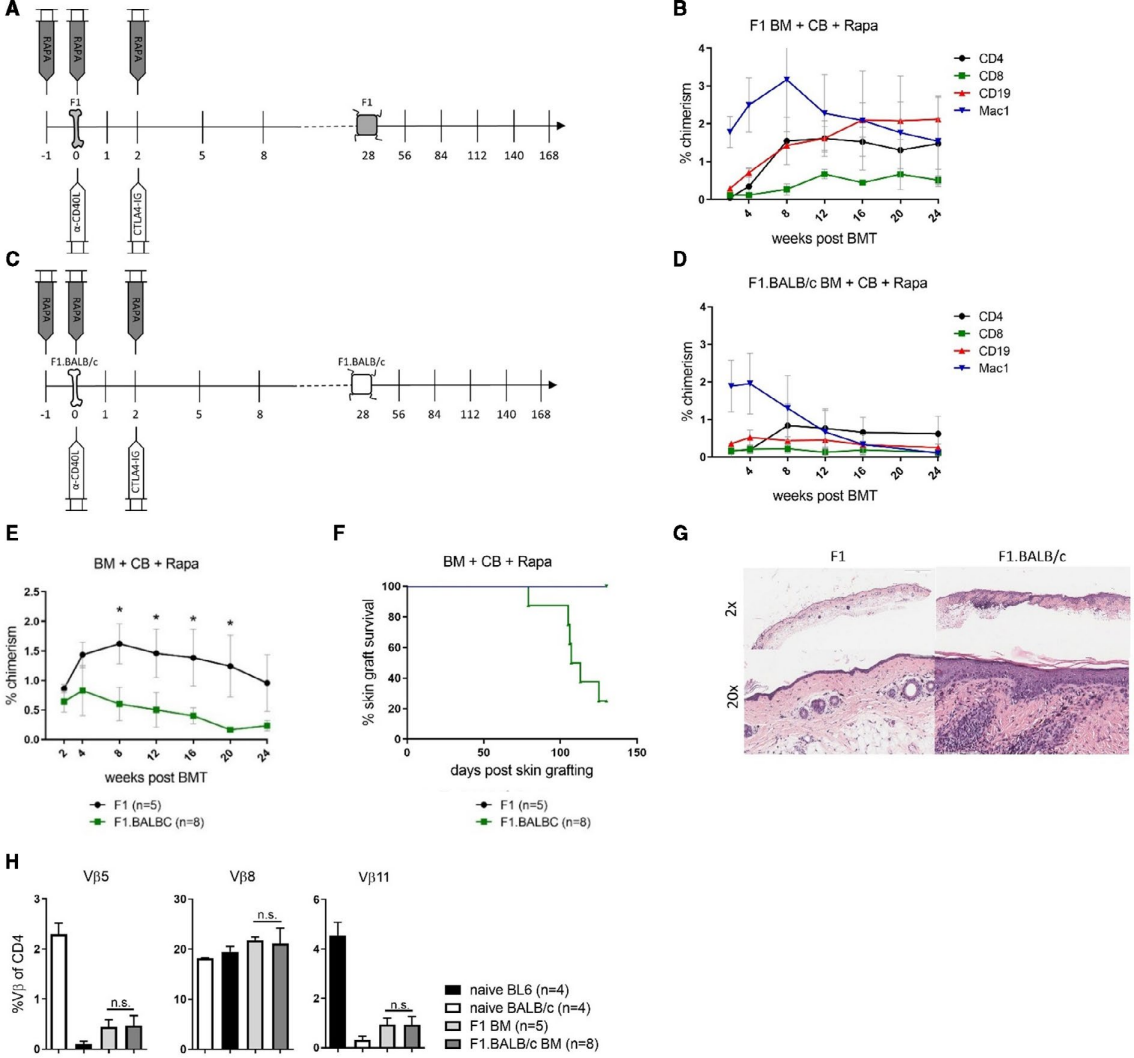
Minor antigen disparities impede skin graft tolerance. C57BL/6 mice received 20 × 10^6^ F1 or F1.BALB/c bone marrow (BM) under costimulation blockade (CB) and rapamycin. A, Schematic illustration showing the course of the experimental protocol of F1 donors. B, Mean percentages ± SD of donor blood chimerism among indicated lineages is shown over time. (n = 5). C, Schematic illustration exhibiting the course of the experimental protocol compromising F1.BALB/c donors. D, Mean percentages ± SD of indicated donor leukocyte populations are shown over time. (n = 8). E, The mean percentages +/‐ SD of total chimerism were compared between recipients of F1 and F1.BALB/c bone marrow transplantation (BMT) recipients overt time. F, Donor skin graft survival of specified BMT recipients is shown over time. G, Representative images of hematoxylin and eosin staining of long‐term surviving skin grafts of F1 and F1.BALB/c BMT recipients. Representative mice are shown. H, The reduction of alloreactive CD4 T cells (Vβ5, Vβ11) was compared in the blood between recipients of F1 and F1.BALB/c BM at the end of the observation period (24 wk after BMT). Bars represent mean ± SD [Color figure can be viewed at wileyonlinelibrary.com]

In agreement with our previous results, transplanting 20 × 10^6^ F1 BM cells into C57BL/6 recipients under the cover of CB and rapamycin (Figure 3A) induced long‐lasting mixed chimerism in multiple lineages (Figure 3B). Moreover, F1 skin allografts survived indefinitely (Figure 3F) and showed little or no signs of cellular rejection on histological inspection (Figure 3G). Transplanting equal numbers of F1.BALB/c BM into C57BL/6 recipients under the same conditions (Figure 3C) likewise resulted in BM engraftment albeit chimerism levels were lower (Figure 3D). Total chimerism levels between both groups were comparable over the first 2 weeks. Thereafter F1.BALB/c chimerism levels hardly increased and remained significantly lower than those of F1 donors (Figure 3E). The majority of BMT recipients (6/8) rejected F1.BALB/c skin allografts by the end of the follow‐up (MST = 111d) (Figure 3F) and the few surviving grafts showed pronounced signs of graft attrition (Figure 3G).

To rule out that the lower chimerism levels could result in a less pronounced deletion of alloreactive CD4 T cells that in turn could lead to shorter survival of skin allografts, we measured distinct superantigen‐reactive Vβ subsets in the blood of BMT recipients as surrogates for donor‐reactive T cells at the end of follow‐up (Vβ5^+^ and Vβ11^+^ CD4^+^ cells recognize superantigens in the context of donor MHC I‐E^20^). The deletion of “donor‐specific” CD4 T cells was far advanced in both groups without showing considerable differences (Figure 3H). Because of the limitations of the experimental system, however, no conclusion with regard to the deletion of mature CD8 cells can be drawn, which likely have a prominent role in the rejection of MiHA‐disparate skin. In summary, these data suggest that MiHA disparities, which are present to a higher degree in F1.BALB/c than in F1 skin allografts, lead to lower levels of chimerism and skin graft rejection despite stable mixed chimerism.

### 3.3 The absence of minor antigen disparities promotes tolerance in mixed chimeras

**FIGURE 4:**
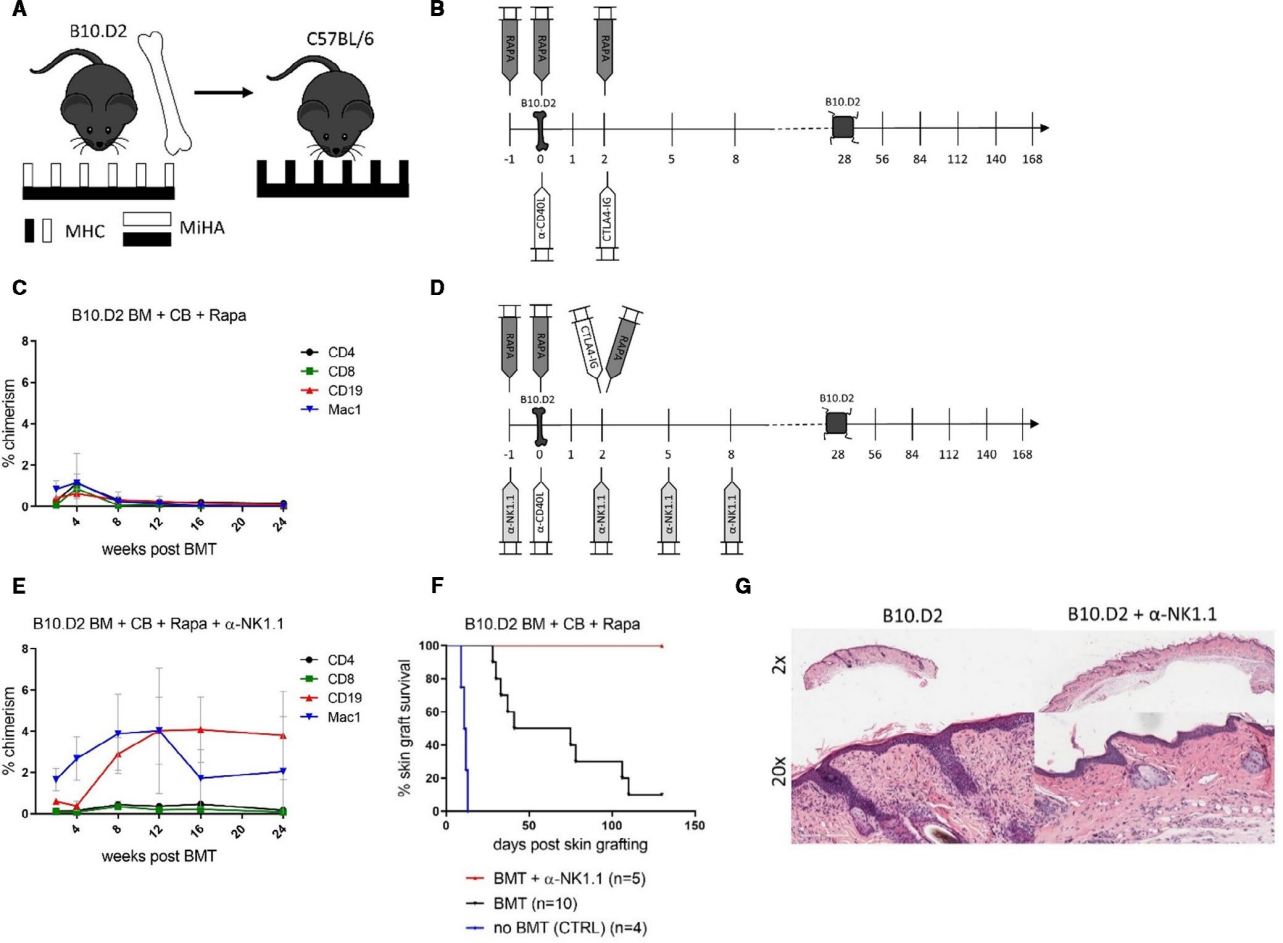
Absence of minor antigen disparities promotes tolerance. C57BL/6 mice received 20 × 10^6^ B10.D2 BM under CB and rapamycin. Selected groups of mice additionally received natural killer (NK) cell‐depleting antibodies (α‐NK1.1) at the time of bone marrow transplantation (BMT). A, Schematic illustration of the experimental setup describing the major (MHC) and minor antigen (MiHA) disparities between donor and recipient. B, Schematic illustration of the experimental protocol employing B10.D2 donors without NK cell depletion. C, The mean percentages ± SD of donor chimerism of indicated blood leukocyte populations is shown over time. (n = 10). D, Schematic illustration of the experimental protocol with B10.D2 donors and transient NK cell depletion. E, The mean percentage ± SD of donor chimerism within indicated blood leukocyte populations of recipients receiving aNK1.1 is shown over time. (n = 5). F, Donor (B10.D2) skin graft survival of specified BMT recipients and naïve recipients (CTRL) is shown over time. G, Representative images of hematoxylin and eosin staining of long‐term surviving skin B10.D2 [Color figure can be viewed at wileyonlinelibrary.com]

To further investigate the role of MiHA as possible cause for the lack of skin graft tolerance in stable mixed chimeras we deployed B10.D2 mice as donors. These mice share the same background with C57BL/6 recipients but express the identical MHC haplotype as BALB/c donors (H2^d^). Consequently, only MHC disparities (without MiHA disparities) exist between donor and recipient (Figure 4A). Transplanting 20 × 10^6^ B10.D2 BM cells into C57BL/6 mice under CB and rapamycin (Figure 4B) was not sufficient to induce long‐term chimerism. Nine out of 10 mice completely lost chimerism by 4 weeks and 1 recipient maintained low levels of mixed chimerism until the end of follow‐up (Figure 4C). All mice rejecting B10.D2 BM also gradually rejected B10.D2 skin allografts over time (MST = 58, *P* < .0001 vs naïve recipients) (Figure 4F). However, if NK cells were depleted transiently at the time of BMT (d–1, d2, d5, d8) by α‐NK1.1 (Figure 4D), all mice (5/5) developed durable levels of mixed chimerism that was markedly pronounced in the B cell and myeloid lineage (Figure 4E). Notably, after NK cell depletion, all chimeras retained B10.D2 skin allografts indefinitely (Figure 4F) without exhibiting histological signs of rejection (Figure 4G). Taken together, these results imply that donor MHC‐reactive NK cells prevent BM engraftment under CB and rapamycin whereas alloreactive T cells targeting MiHAs disparities prevent skin graft tolerance in mixed chimeras.

### 3.4 Minor antigens impede tolerance in the absence of MHC disparities

**FIGURE 5:**
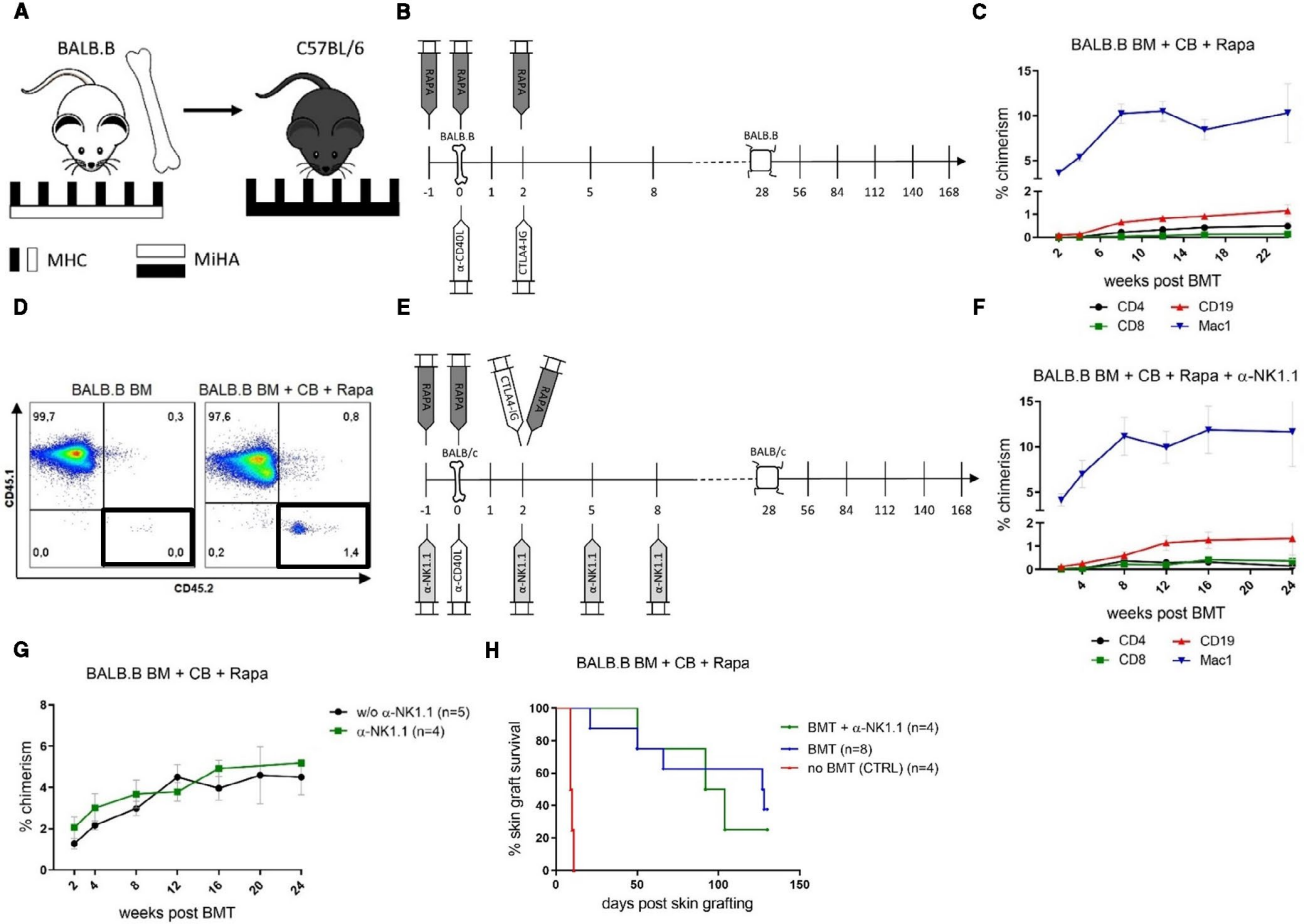
Minor antigens provoke skin allograft rejection in the absence of major histocompatibility complex (MHC) disparities. C57BL/6 mice received 20 × 10^6^ BALB.B BM under costimulation blockade (CB) and rapamycin. Selected groups of mice additionally received natural killer (NK) cell‐depleting antibodies (α‐NK1.1) at the time of bone marrow transplantation (BMT). A, Schematic illustration of the experimental setup outlining the major (MHC) and minor antigen (MiHA) disparities between donor and recipient. B, Schematic illustration of the experimental protocol employing BALB.B donors without NK cell depletion. C, The mean percentage ± SD of donor chimerism within indicated blood leukocyte populations is shown over time (n = 8). D, Dot plot shows donor (CD45.2) and recipient (CD45.1) blood cells in representative mice 2 wk post‐BMT. E, Schematic illustration of the experimental protocol of BALB.B donors and transient NK cell depletion. F, The mean percentage of donor chimerism within indicated blood leukocyte populations in recipients of a‐NK1.1 is shown over time (n = 4). G, The mean percentage ± SD of total blood chimerism over time with and without a‐NK1.1. H, Donor (BALB.B) skin graft survival of specified BMT recipients or naïve recipients (CTRL) is shown over time [Color figure can be viewed at wileyonlinelibrary.com]

To assess the role of isolated MiHAs disparities in the absence of MHC disparities, we employed BALB.B mice as donors that express the same MHC haplotype as C57BL/6 recipients albeit on the background of BALB/c mice (Figure 5A). To distinguish donor from recipient cells, we transplanted 20 × 10^6^ CD45.2^+^ BALB.B BM cells into CD45.1^+^ C57BL/6 recipient mice. In this setting, CB and rapamycin (Figure 5B) were sufficient to achieve durable mixed chimerism in all mice (8/8) (Figure 5C). In comparison, no donor cells could be recovered 2 weeks after BMT if CB and rapamycin were omitted (Figure 5D). However, despite successful BM engraftment 5 out of 8 mice gradually rejected BALB.B skin grafts (MST = 127.5 vs MST = 9.5 in untreated controls) (Figure 5H). Depleting NK cells at the time of BMT (d–1, d2, d5, d8) (Figure 5E) did not affect levels of chimerism (Figure 5F,G) and did not improve BALB.B skin allograft survival (MST = 98) (Figure 5H). Accordingly, we conclude that MiHAs do not hamper BM engraftment under CB and rapamycin but impede the induction of skin graft tolerance.

## 4 DISCUSSION

Here we investigated the role of MHC and MiHA antigen barriers for the induction of chimerism and the establishment of skin graft tolerance under noncytotoxic recipient conditioning. Collectively, our data provide evidence that in nonirradiated recipients solely conditioned with CB and rapamycin, alloreactive NK cells, triggered by MHC but not by minor disparities, are the major barrier for BM engraftment, whereas T cells targeting (skin specific) MiHA are the major factor impeding donor skin graft tolerance.

Because the time of skin graft rejection coincided with the return of NK cells after transient antibody depletion, we first ruled out that the returning NK cells trigger rejection by extending NK cell depletion in mixed chimeras which, however, did not improve skin graft survival. Subsequently the question arose why chimerism persisted long term while skin allografts were gradually rejected. This phenomenon, known as split tolerance, has not yet been fully clarified. Several factors have been proposed to favor the occurrence of split tolerance. Tissue‐specific expression of MiHAs and minimal conditioning protocols that keep the endogenous T cell compartment largely intact are both currently considered as prime factors.^21^ The marked difference between the survival of BALB/c and B10.D2 skin allografts that we observed in NK cell‐depleted recipients strongly supports this hypothesis. In both cases, durable chimerism emerged after NK cell depletion but skin grafts only survived in the absence of MiHAs disparities (B10.D2 → C57BL/6). Because durable chimerism levels were achieved with both donors, it seems reasonable to assume that MiHAs expressed by BALB/c but not B10.D2 resulted in different skin survival rates. The role of MiHAs for tolerance induction has often been neglected in early tolerance studies because most donors were MHC disparate but expressed the same MiHAs as the recipients.^12^, ^22^ We have recently addressed this issue and found that the absence of MiHA promotes chimerism and tolerance in mice receiving nonmyeloablative TBI and CB. In contrast to the current experimental setup, these mice did not receive rapamycin and showed significantly higher levels of mixed chimerism due to nonmyeloablative TBI.^23^

We also recently found that adding regulatory T cells from the recipient to the donor BM transplant promotes MiHA tolerization under CB and rapamycin in nonirradiated mice. The role of MiHAs for the induction and maintenance of chimerism, however, was not directly addressed in this study. The exact mechanisms are not yet known, but regulatory mechanisms seem to prevail over deletional mechanisms with regard to MiHA tolerization.^24^ One major difference to the present study is the use of a cellular therapy instead of cell‐depleting antibodies. Antibody‐dependent cellular cytotoxicity is accompanied by the release of inflammatory cytokines that might interfere with tolerization mechanisms. However, BALB.B skin grafts were also rejected in the absence of NK cell‐depleting antibodies. Therefore, we rather assume that indirectly alloreactive T cells are responsible for the late allograft loss.

The role of MiHAs as the main reason for the lack of tolerance is further supported by the decreased survival rates of F1.BALB/c skin grafts in comparison to F1 donors (both express the same MHC molecules but different MiHAs). In contrast to recipients of BALB/c grafts, NK cells of F1 recipients were not depleted. This raised the question whether recipient NK cells could contribute to the rejection of F1.BALB/c skin grafts. MiHAs can reinforce the acute rejection of solid allografts through the release of proinflammatory cytokines by NK cells.^25^ Missing self‐recognition can trigger chronic vascular rejection of solid organ transplants^26^ but it remains elusive so far whether MiHAs alone can induce graft damage.

What speaks against the contribution of NK cells is that extending NK cell depletion did not improve BALB/c skin graft survival and that F1.BALB/c grafts survived in NK cell intact mice as long as BALB/c skin grafts in NK cell‐depleted mice (both share the same MiHAs).

Therefore, we conclude that skin‐specific MiHAs of F1.BALB/c skin grafts primarily elicit indirect T cell alloresponses that cause late graft rejection. However, we cannot rule out the alternative possibility that MiHAs inherited from C57BL/6 might offer a survival advantage to transplants from F1 mice by a—yet undefined—inhibitory mechanism.

Our recent data showed that NK cells play a major role in BM rejection in nonirradiated mice under CB and rapamycin.^18^ We could extend our previous results by showing that MHC disparities are the main driver for NK cell‐mediated BM rejection whereas MiHAs only play a minor role since neither BALB/c (H2^d^) nor B10.D2 (H2^d^) BM engrafts in C57BL/6 (H2^b^) recipients treated with CB and rapamycin unless NK cells are depleted (whereas BALB.B BM does engraft without NK depletion). Tissues from both donors are prone to NK cell attack because of the absence of recipient MHC molecules (H2^b^) on their surface.^27^ In turn, BM from F1 (H2^b/d^), F1.BALB/c (H2^b/d^), and BALB.B (H2^b^) donors—none of which are targets for recipient NK cells—readily engrafted under CB and rapamycin. In line with this, it could already be shown in irradiated recipients that NK cells mediate the early elimination of MHC mismatched hematopoietic cells.^15^ Our study extended these observations to nonirradiated recipients in whom NK cell‐mediated rejection is qualitatively and quantitatively different.^28^ Another study also demonstrated that NK cell depletion can overcome CB resistant BM rejection. At this, a nonengrafting dose of donor BM (20 × 10^6^) was given (d0) before the recipients received a nonmyeloablative dose of busulfan (d5) together with a second donor BM dose (d6) and an extended course of CB. NK cell‐depleted recipients displayed multilineage chimerism and retained skin allografts long term.^29^ In contrast to our study, NK cell‐depleted mice exhibited high levels of donor chimerism (~50%) because of the nonmyeloablative pretreatment, which again underlines that rejection seems to be significantly different in the absence of cytotoxic treatment. It should also be noted that BALB/C skin grafts survived longer under CB and rapamycin if low doses of irradiation were used instead of NK cell depletion.^13^

However, the differences between F1 and F1 BALB/c donors suggest that MiHAs may influence donor chimerism levels at later time points. Vice versa, the absence of MiHA disparities has been shown to increase chimerism levels in a nonmyeloablative BMT model.^23^ The expression of NKG2D ligands on certain donor strains can reinforce NK cell‐mediated BM rejection through missing self‐recognition.^30^ Because F1 donors are not subjected to NK cell alloreactivity we, however, suggest that rather indirect alloreactive T cells account for the differences between F1 and F1.BALB/c donor chimerism levels.

In summary, this study provides further insight into the mechanisms that induce and maintain tolerance through mixed chimerism and delineates the distinct roles of MHC and MiHA barriers in the noncytotoxic setting.

## DISCLOSURE

The authors of this manuscript have no conflict of interests to disclose as described by the *American Journal of Transplantation*.

## Data Availability

Data available on request from the authors.
